# Remarks on the Cow-Pox

**Published:** 1799-08

**Authors:** John Ring

**Affiliations:** Member of the Corporation of Surgeons


### *Remarks on the Cow-Pox.*

[Communicated by Mr. John Ring, Member of the Corporation of Surgeons.]

### *To the Editors of the Medical and Phyſical Journal.*

Gentlemen,

THE introduction of the cow-pox into practice, as a ſubſtitute for the ſmall-pox, at preſent engages a conſiderable ſhare of the attention of medical men. Permit me, therefore, to inſert in your very valuable publication, a few remarks on that diſeaſe.

Great praiſe is due to Dr. Jenner, for this improvement; and while I join in paying a juſt tribute of applauſe to his merit, I ſincerely hope, that the that the preventive he propofes will prove fuccefsful, and at length exterminate one of the moft dreadful fcourges of the human race.

Of thofe whom I have inoculated, or feen inoculated, with vaccine matter, few have had any confiderable eruption, and thofe few were inoculated with matter, which there is reafon to believe was not taken from the original puftule on the arm; a circumftance which Dr. Woodville has proved to be of great confequence in this difeafe.—The reft fcarcely appeared to labour under the leaft indifpofition, except what arofe from the inflammation of the arm; and even that was not worfe than in cafes of inoculation with variolous matter.

I have feen, at the Medical Society, a letter which was received by Dr. Jenner from a furgeon in the country, giving an account of his having inoculated above a hundred perfons, with vaccine matter fent him by Dr. Jenner; and that only two or three had any puftules, which were few, and confined to the arm.

That eruptions, in this difeafe, are not peculiar to the metropolis, as Dr. Jenner fuppofes, other inftances prove, befides thofe recorded by Dr. Woodville. The only two patients inoculated by me, who had many puftules, refided at Kenfington till after the eruption had taken place; and the only one befides, whom I have feen, at Highbury place. A phyfician of Bath informed me, that he had inoculated two, both of whom had eruptions; and it is a little remarkable, that the matter of all the patients above mentioned came from a practitioner, who was in the habit of taking it from the fecondary puftules; a circumftance which I have carefully avoided.

The fuccefs of the practice has, on the whole, been fuch as to gratify every reafonable expectation; efpecially if allowance be made for the error of taking the matter from an improper puftule; an error eafy to be avoided in future. Had all the patients inoculated with vaccine matter at the Small-pox Hofpital, by Dr. Woodville, been in the houfe, and under his immediate care, it is probable his firft report would have been ftill more favourable: but even from that, I fhould not hefitate to prefer the vaccine to the variolous difeafe.

In the fhort time which has elapfed fince I began to write thefe remarks, I have heard of two families, plunged in the deepeft affliction by the inoculated fmall-pox; while, on the other hand, only one folitary inftance is on record, of the cow-pox proving fatal; and we have reafon to believe, from Dr. Woodville's fubfequent report, that even that unfortunate event would not have happened, if the difeafe had then been as well underftood as it is at prefent. Be that as it may, I hope no practitioner will in future inoculate late with any vaccine matter but what is taken from the original puſtule, unleſs he thinks proper to inoculate himſelf. I deem it alſo a duty, in this age of experiment, to caution medical men not wantonly to expoſe the lives of their fellow creatures to any unneceſſary danger; and not to inoculate with one kind of matter, till another has produced its final effect.

That two morbid actions cannot take place in the body at the ſame time, may paſs uncontradicted in the ſchools, but not in the field of experience. One caſe of a complication of the ſmall-pox and the meaſles, was read before the Medical Society; and others I could prove by the moſt reſpectable teſtimony.

Doctor Moseley, in his treatiſe on Sugar, lately publiſhed, expreſſes a ſuſpicion, that the cow-pox, can only render the habit inſuſceptible of the ſmall-pox "*for a time.*"—This is refuted by volumes of evidence, and a cloud of witneſſes.—He ſays, "Inoculation has diſarmed the ſmall-pox of its terrors."—This is refuted by the whole world.

He aſſerts, that "accidents in the inoculated ſmall-pox are uncommon." Would to God experience did not diſprove that aſſertion, and convince practitioners in general, that no care, no ſkill ever did, or ever can, tame that dreadful hydra—the ſmall-pox!

He tells us, "We all know, from experience, that diſeaſe, properly treated, leaves nothing after it injurious to the conſtitution."—That we do not all know it, is certain: if Dr. Moſeley has been ſo happy as to diſcover the ſecret, I hope his humanity will prompt him to diſcloſe it.

It is well known, that the ſmall-pox, whether natural or inſititious, is one of the moſt common cauſes of ſcrofula; and my experience leads me to believe, that the abſurd cuſtom of giving cathartics after this and other eruptive diſorders, by debilitating the habit, augments their tendency to produce that horrid diſeaſe.

Dr. Moſeley tell us, "he wiſhes not to diſcourage inquiry," and admits, that "the object well deſerves it;" yet, with ſome degree of inconſiſtence, he adds, that he wiſhes "to guard parents againſt ſuffering their children becoming *victims to experiment*."—My wiſhes are not leſs ardent than his:—he wiſhes to prevent children from becoming victims to *experiment*; I wiſh to prevent them from becoming *victims to the ſmall-pox*.

Dr. Moſeley intended his eccentric remarks, which are introduced rather mal-a-propos in a Treatiſe on Sugar, as an antidote for what he calls the *Cow-mania*.—He himſelf ſeems to labour under the *Cow-phobia*.—He aſks, if any any perſon can ſay, " what may be the conſequences of introducing a *beſtial bumour* into the human frame, after a long lapſe of years?"—I beg leave to aſk, in my turn, if any perſon can ſay, what may be the conſequences, after a long lapſe of years, of introducing into the human frame, *cows' milk*, *beef-ſteaks*, or *a mutton-chop?*

I hope medical men will in future be cautious, how they prejudice the public mind againſt a fair trial of a practice, warranted by obſervation, and recommended by a phyſician of diſtinguiſhed abilities; and not ſpread a ſerious alarm, where even the vulgar and illiterate, who are generally moſt averſe to all innovations, and of courſe to all improvement in the practice of phyſic, have not hitherto hinted a ſuſpicion.

Dr. Moſely argues, as if the cow-pox were a new diſeaſe in the human ſpecies; a ſuppoſition which it is unneceſſary to refute. One of the advantages propoſed by Dr. Jenner and Dr. Pearſon, from the eſtabliſhment of the new practice, is, that although the diſorder in queſtion is ſo common, and has long been well known in many parts of the kingdom, *it never has been ſuſpected to leave behind it any other diſeaſe.*

I am happy in being able to add my teſtimony to that of Drs. Jenner, Pearſon, and Woodville, in confirmation of the efficacy of the new practice; having inoculated with variolous matter, twelve perſons whom I had previouſly inoculated for cow-pox, all of whom eſcaped the infection of the ſmall-pox.

Since moſt of the foregoing obſervations were written, I have ſeen Dr. Woodville's ſecond report, confirming the opinion he expreſſed in the firſt; and acknowledging that he has lately been much more ſucceſsful in his practice; in conſequence of refraining from taking matter from patients who had the diſeaſe ſeverely—a caution I have always obſerved.

It has been aſſerted, that the cow-pox cannot be communicated but by contact; and in the moſt poſitive manner, that it certainly cannot be communicated by means of effluvia, where there is no puſtule but that of the arm. In this reſpect, I think, gentlemen have been rather too haſty in forming their concluſions, when the diſorder has been ſo ſhort a time under their immediate care and inſpection. I have ſeen one inſtance, where the diſeaſe was communicated without a poſſibility of its being received by contact; and where the child, from whom the infection came, had no puſtule but that on the arm; and from good authority I have heard of another inſtance, where the infection was caught from one who had a conſiderable eruption.

Dr. Jenner

Dr. Jenner, having found ſome difficulty in communicating the infection of cow-pox, propoſes a method which I think rather tedious and troubleſome. I beg leave therefore to ſuggeſt, that if, inſtead of puncturing or ſcratching the ſkin in the ordinary way, the lancet, after liquifying the matter, if neceſſary, by ſteam, is laid almoſt flat upon the ſkin, and then inſerted obliquely, ſo as to raiſe the cuticle, moving the point backwards and forwards a few times, to ſeparate the matter from the lancet, and wiping it on the puncture,—the matter will in general be received and retained by the cuticle, as by a valve; and abſorption be rendered almoſt certain.

The inflammation of the arm being a circumſtance worthy of the attention of medical practitioners, I preſume to recommend a ſimple poultice, to be applied cool; and, if any ulceration ſhould take place and produce a ſinus, a little lint, dipped in melted ung. ceræ, to fill up the cavity, and a poultice to be applied over. This plan I have never known to fail, in the worſt caſes I have ever ſeen.

Allow me to add to the preceding remarks, that the method of extinguiſhing quickſilver by means of ſtale lard, as propoſed by Mr. Proctor, was communicated to me by the late Mr. Wyatt, eight or nine years ago. He told me he had lard about twenty years old, which he uſed for that purpoſe.

The caſe of a perſon who loſt his life by unſkilful bleeding, inſerted in your laſt number, is deſervedly recorded *in terrorem:* but I cannot agree with your correſpondent, Dr. Vaughan, of Rocheſter, that the fatal cataſtrophe could be occaſioned by the juices of another perſon in health. I am rather inclined to attribute the melancholy event to the length of the orifice, and to a neglect of cloſing it properly, and promoting union by the firſt intention: hence inflammation and every other ill conſequence, too often, alas! occaſioned by the tribe of ignorant phlebotomiſts, and other charlatans, who are ſuffered in this country to wield the *inſtruments of death* with impunity.

New-street, Hanover-square,

*July* 6, 1799.

